# Supplementary material for: LncRNA SChLAP1 promotes cancer cell proliferation and invasion via its distinct structural domains and conserved regions
Source: bioRxiv. 2025 Jan 28:2025.01.28.635288. Preprint. [Version 1] doi: 10.1101/2025.01.28.635288 (PMC11838354; doi:10.1101/2025.01.28.635288)
Supplement: 1 [file NIHPP2025.01.28.635288V1-supplement-1.pdf]

bioRxiv preprint doi: https://doi.org/10.1101/2025.01.28.635288; this version posted January 28, 2025. The copyright holder for this preprint (which was not certified by peer review) is the author/funder, who has granted bioRxiv a license to display the preprint in perpetuity. It is made available under aCC-BY 4.0 International license.

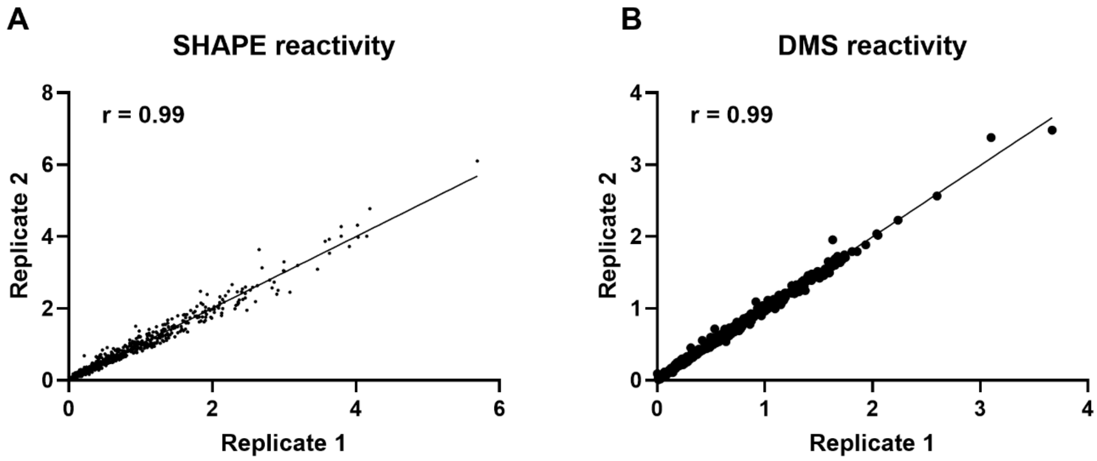

Supplementary Figure S1. Chemical probing of SChLAP1 in 25 mM Mg^{2+} folding buffer. SHAPE reactivity (A) and DMS reactivity (B) results were reproducible among different biological replicates. Pearson correlation coefficient (*r*) values are indicated.

bioRxiv preprint doi: https://doi.org/10.1101/2025.01.28.635288; this version posted January 28, 2025. The copyright holder for this preprint (which was not certified by peer review) is the author/funder, who has granted bioRxiv a license to display the preprint in perpetuity. It is made available under aCC-BY 4.0 International license.

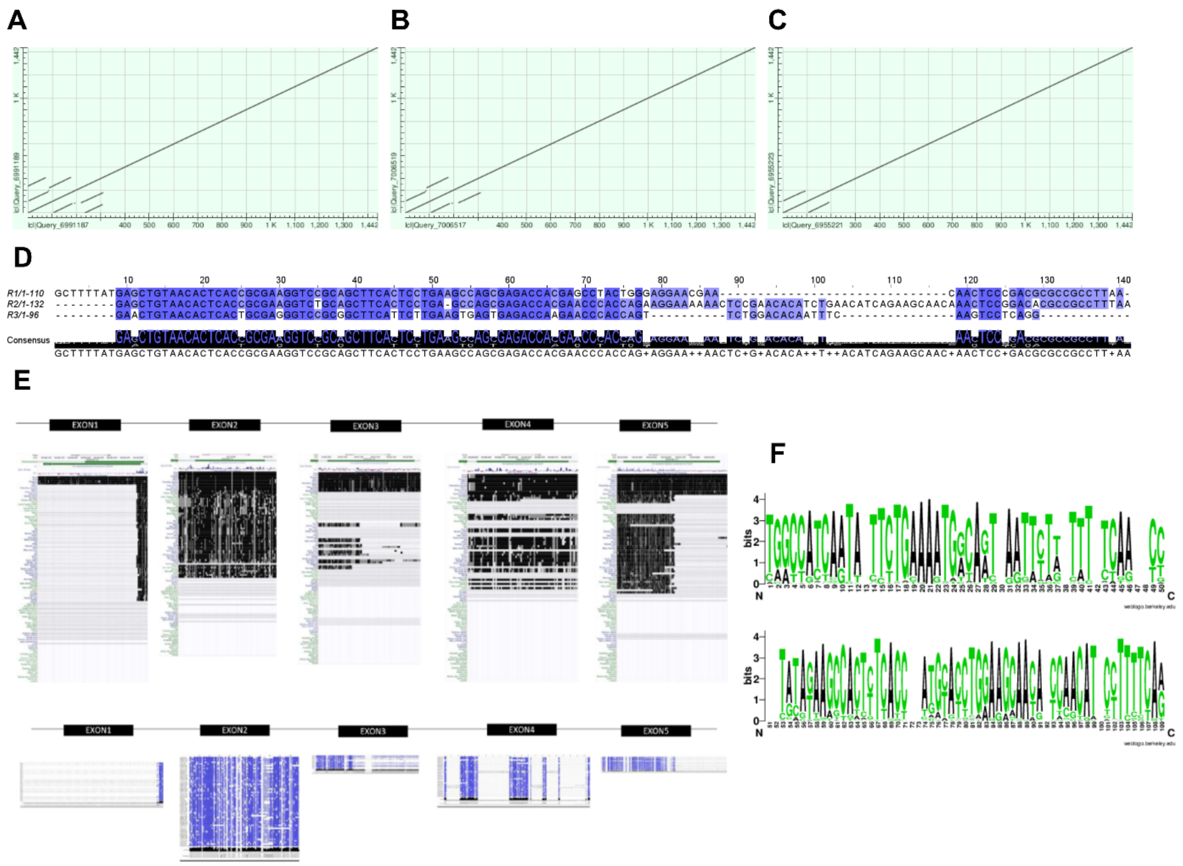

Supplementary Figure S2. Dot plots of BLAST self-alignment of SChLAP1 sequences with a setting of “somewhat similar (A),” “more dissimilar (B),” and “highly similar (C)”. Comparison of the SChLAP1 sequence with itself revealed the presence of several repetitive sequences with varying lengths and similarities. (D) Multiple sequence alignment by Clustal Omega revealed that the first exon of SChLAP1 consists of three repeat regions, including Repeat 1 (R1), Repeat 2 (R2), and Repeat 3 (R3) (50). (E) Multiz alignment (top) and FASTA sequence alignment (bottom) for each exon. Black- and blue-colored regions represent sequence conservation. (F) Graphical representation of conserved nucleic acid sequences of SChLAP1. Multiple highly conserved motifs were identified throughout the second exon: ‘TGGCCATCAATA’ from Nucleotides 1-12, ‘TTCTGAAAATGGCAGT’ from Nucleotides 14-29,

bioRxiv preprint doi: https://doi.org/10.1101/2025.01.28.635288; this version posted January 28, 2025. The copyright holder for this preprint (which was not certified by peer review) is the author/funder, who has granted bioRxiv a license to display the preprint in perpetuity. It is made available under aCC-BY 4.0 International license.

‘TATAGAAGCCACTCTCACC’ from Nucleotides 53-71 and ‘ATGCACCTGGAAGCAACA’ from Nucleotides 74-91. The height of each stack represents the sequence conservation.

bioRxiv preprint doi: https://doi.org/10.1101/2025.01.28.635288; this version posted January 28, 2025. The copyright holder for this preprint (which was not certified by peer review) is the author/funder, who has granted bioRxiv a license to display the preprint in perpetuity. It is made available under aCC-BY 4.0 International license.

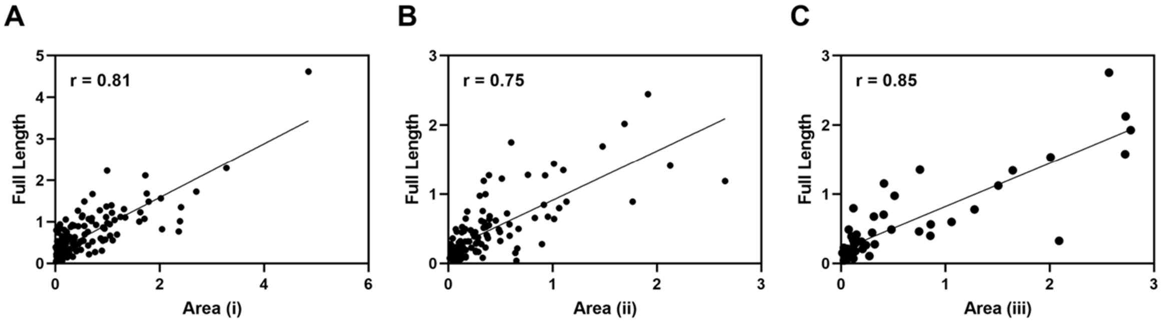

Supplementary Figure S3. (A-C) Scatter plots comparing SHAPE reactivities of Area (i) through (iii) (see Figure 5) with the corresponding region in the full-length SChLAP1. The data represent the average of two biological replicates. Pearson correlation coefficient (*r*) values are indicated.

bioRxiv preprint doi: https://doi.org/10.1101/2025.01.28.635288; this version posted January 28, 2025. The copyright holder for this preprint (which was not certified by peer review) is the author/funder, who has granted bioRxiv a license to display the preprint in perpetuity. It is made available under aCC-BY 4.0 International license.

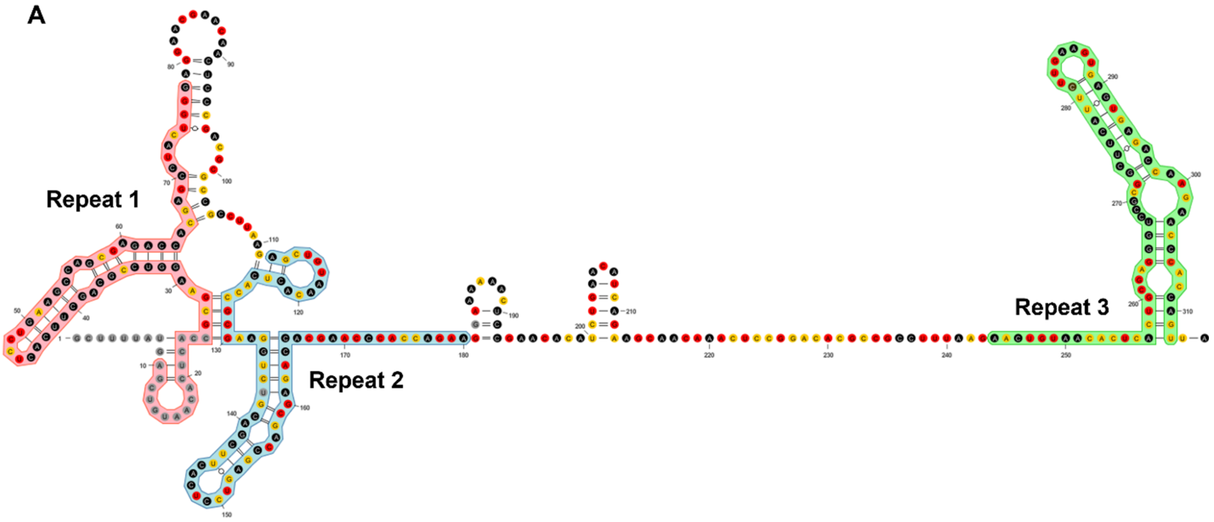

Supplementary Figure S4. Sequential and structural analysis of the repeats. Each of the three repeats is highlighted using different color schemes.

bioRxiv preprint doi: https://doi.org/10.1101/2025.01.28.635288; this version posted January 28, 2025. The copyright holder for this preprint (which was not certified by peer review) is the author/funder, who has granted bioRxiv a license to display the preprint in perpetuity. It is made available under aCC-BY 4.0 International license.

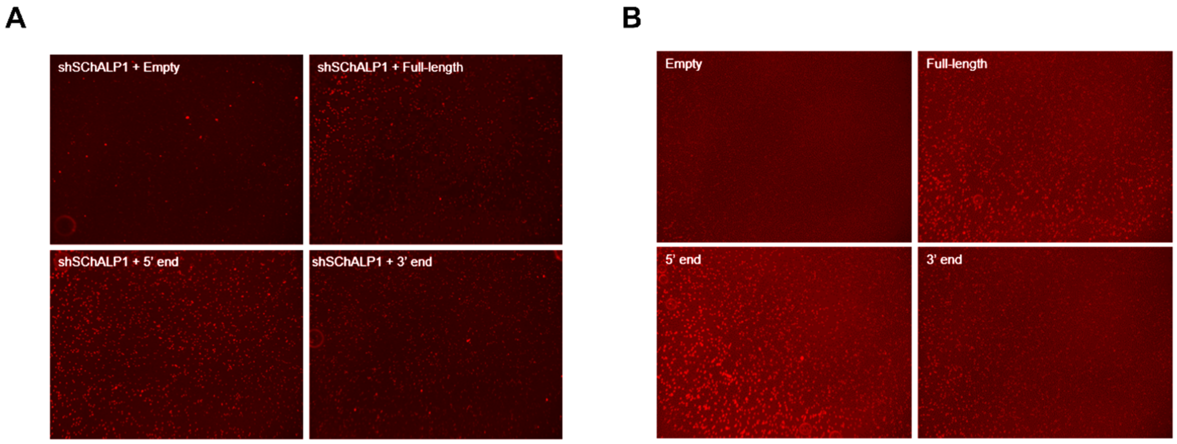

Supplementary Figure S5. Representative images of transwell invasion assays in LNCaP (A) and 22Rv1 cells (B). Cell invasion was assessed by averaging data from three randomly selected fields.

bioRxiv preprint doi: https://doi.org/10.1101/2025.01.28.635288; this version posted January 28, 2025. The copyright holder for this preprint (which was not certified by peer review) is the author/funder, who has granted bioRxiv a license to display the preprint in perpetuity. It is made available under aCC-BY 4.0 International license.

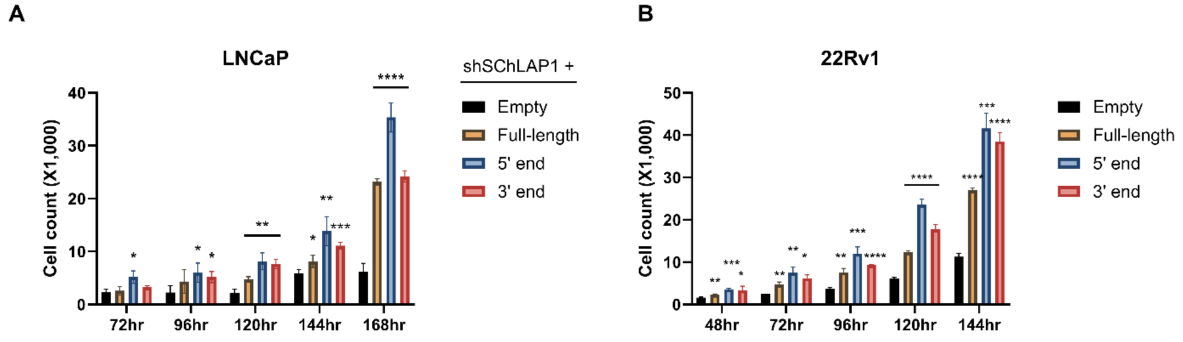

Supplementary Figure S6. Cell proliferation was measured by counting cells upon overexpressing the 5' or 3' end of SChLAP1 in LNCaP (A) and 22Rv1 cells (B). These plots are bar graphs depicting the data shown in Figures 6H and 6I.

bioRxiv preprint doi: https://doi.org/10.1101/2025.01.28.635288; this version posted January 28, 2025. The copyright holder for this preprint (which was not certified by peer review) is the author/funder, who has granted bioRxiv a license to display the preprint in perpetuity. It is made available under aCC-BY 4.0 International license.

Supplementary Table S1. Primer sequences used in this study.

**Site-directed mutagenesis**		
**Target**	**Forward Primer**	**Reverse Primer**
Fragment 1 (Nucleotides 222-651)	GACACGCCGCCTTTAAGAAC	CTTTCTCCATCCCGCTCCTC
Fragment 2 (Nucleotides 956-1428)	TCCATCAAGTCCTGGCTAACC	AATGGGCTCACAGTTCCACG
pLenti-GIII-3'-end	CTGAATTTCCATCAAGTCCTGG C	ATTTATAAGTGAAAGAGGTTTAAT GG
***in vitro* chemical probing combined with mutational profiling**		
**Target**	**Forward Primer**	**Reverse Primer**
Full-length SChLAP1	GCTTTTATGAGCTGTAAC	ATTTATAAGTGAAAGAGGTTTAAT GG
Fragment 1 (Nucleotides 222-651)	GACACGCCGCCTTTAAGAAC	CTTTCTCCATCCCGCTCCTC
Fragment 2 (Nucleotides 956-1428)	TCCATCAAGTCCTGGCTAACC	AATGGGCTCACAGTTCCACG
***in vivo* chemical probing combined with mutational profiling**		
**Target**	**Forward Primer**	**Reverse Primer**
Nucleotides 1-740	GCTTTTATGAGCTGTAAC	GCCTCTTGGGTTCACCATCT
Nucleotides 586-1119	CCTCCCTGAAGAAGCTGAATAT C	CACAGCCAAACCATATCACATG
Nucleotides 958-1436	CTGAATTTCCATCAAGTCCTGG C	ATTTATAAGTGAAAGAGGTTTAAT GG
**RT-qPCR**		
**Target**	**Forward Primer**	**Reverse Primer**
Full-length SChLAP1	TGGACACAATTTCAAGTCCTC	CATGGTGAAAGTGCCTTATAC
5'-end SChLAP1	TGGACACAATTTCAAGTCCTC	CATGGTGAAAGTGCCTTATAC
3'-end SChLAP1	ACCAATGTTCACTGTGAAGGA	GGGGACACAGCCAAACCATA
GAPDH	GAAGGTCGGAGTCAACGG	ACATGTAAACCATGTAGTTGAGGT
MMP9	CGTCGGTCCGTCCGCTA	GGGAACATCCGGTCCACCT
MMP14	AGTGGATAGCGAGTACCCCA	TTCTCTCAGCGACACCTTCC
VEGF	CCTGTTCCGAGGTTGCCCT	AGGACCAACAGCCACTATGAG
